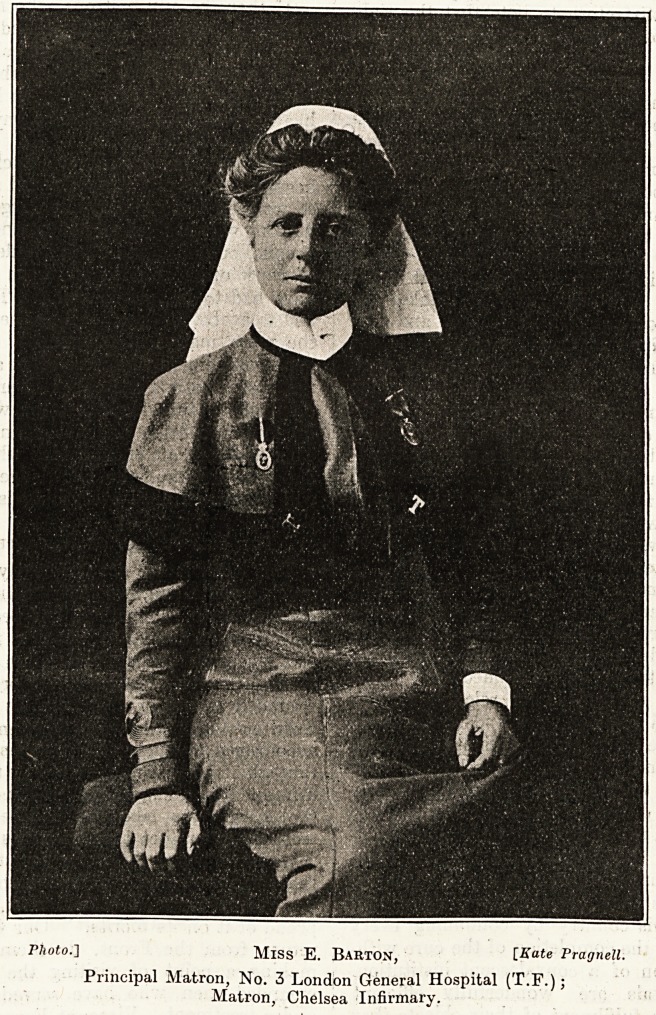# Matrons and Sisters

**Published:** 1916-02-12

**Authors:** 


### THE DEPARTMENT OF NURSING.

### Matrons and Sisters.

Our remarks last week referring to the position of matrons and sisters in civil hospitals which have admitted wounded sailors and soldiers have called forth expressions of gratitude and thanks. One correspondent writes that no one but those who know the working of a hospital can realise what it has been to matrons and sisters in civil hospitals during the last sixteen months, with the senior members of the staff withdrawn for military work. In the great general hospitals, where there are large staffs, in some of which there is also a private staff as a possible reserve, the work has been difficult enough, but in the smaller hospitals it has proved at times burdensome to a degree, especially where the number of senior nurses is so small that sisters have to be appointed from outside. These remarks apply to the majority of hospitals, at any rate, since the war, owing to the large number of excellent sisters who have been withdrawn for military and other duties elsewhere. It speaks volumes for the excellent spirit of those responsible that by continuous devotion on the part of the matrons and their assistants the work has invariably been done, and well done too.

Mr. Arthur Stanley's proposal to establish a College of Nursing seems to be making its way with giant strides, owing to the wise policy of not launching a cut-and-dried scheme at the outset. Mr. Stanley has formulated an outline, has placed himself in communication with and has invited the criticism and help of everybody interested in nursing, as well as those most competent to deal with the proposal on its merits. We hope that those of our readers who are interested in the proposed College—and who is not?—will send us any criticisms or suggestions which are apposite, with a view of improving the scheme and making it fulfil, to the maximum extent, the requirements of modern nursing and the just aspirations of the wisest heads in the nursing profession. Mr. Stanley is anxious that Poor-Law nursing should occupy its proper place in the College scheme, a fact which at first seemed in doubt owing to an accident whereby some Poor-Law matrons and superintendents appear not to have received a copy of Mr. Stanley's circular-letter. This oversight has been rectified, and we are indebted to a correspondent for having pointed it out. The way to help the College of Nursing is to ask questions and not to accept any statement without referring it to Mr. Stanley, or at least writing to us for an explanation. One statement that Mr. Stanley is working in the interests of the untrained nurse is absurd on the face of it, and all

*Photo.*] Miss E. Barton, [*Kate Pragnell.*
Principal Matron, No. 3 London General Hospital (T.F.);
Matron, Chelsea Infirmary.

sensible people will treat it with the contempt it deserves.

A Matron's Functions.

Hospital matrons nowadays have won for themselves a recognised place and authority in the hospital world under the voluntary system. Here and there attempts are still made by ignorant people and local politicians, in the case of hospitals and kindred institutions supported by, or under the control of, municipal, county, and local authorities, to undermine this authority. In this connection it is satisfactory to note that the right of a hospital matron to appoint a nurse was vindicated recently at a meeting of the Bangor City Council, when a recommendation was brought forward that the matron of the Borough Infectious Hospital should open the applications for the post of staff nurse at the institution, make her own selection, and report to the sanitary committee. One Councillor opposed this proposal on the ground that it was contrary to democratic government. He moved that the matron should make the appointment in conjunction with the committee. The medical men on the Council urged that the matron had the right to make the appointment without any interference, and Dr. Thomas properly insisted that the position of the matron would be untenable if there was any such interference with her rights as the Councillor demanded. The matron of every properly constituted hospital was entrusted with the responsibility of making the selection, for she had a greater knowledge of nurses than it was possible for members of such a committee to possess. It is satisfactory to report that an overwhelming majority of the Bangor City Council empowered the matron in question to make the appointment.

The war has tended in many directions greatly to increase the interest taken by the general public in our hospitals throughout the country. The military and Territorial hospitals which are devoted entirely to the reception of the wounded have, as a paramount object, to provide for relaxation, adequate exercise, and outdoor work and sports, with a view to enable the convalescent patients to recover their full health and maintain themselves in good condition, so as to enable them to return perfectly fit to their military duties. In this way the military hospitals, of which those that occupy asylum buildings converted for the purpose are the best type, owing to the amount of open space with extensive lawns and gardens they possess, represent a new hospital type in this country by combining every accommodation for the completion of the cure without the intervention of a convalescent institution. Some war hospitals are wonderfully efficient and attractive. In fulfilment of their objects they have shown splendid enterprise by preparing and publishing each month a gazette or magazine. Several of them have been illustrated by artists of great ability, and the contents and general appearance of these magazines reflect the greatest credit upon everybody concerned. Any hospital which is ambitious of following this excellent example may obtain full information to help them in their project by obtaining a copy of The Hospital of December 18 last, which contains on page 257 an article entitled "Every Hospital's Own Gazette." They might also write to the publishers of The Hospital, 28 and 29 Southampton Street, Strand, London, W.C., enclosing 4d. in halfpenny stamps, asking them to forward a copy of the *Gazette* of the 3rd London General Hospital, Wandsworth, which is always one of the best.

How to Get Help in the Laundry.

If there be any matrons who have found a difficulty in obtaining laundry hands it may interest them to have their attention called to the letter from a high official we publish in the Editor's Letter-Box this week, showing how one hospital has been enabled to overcome any obstacles of the kind.

The matrons and sisters attached to Territorial hospitals have often experienced a superabundance of work in the ordinary discharge of their duties. Anyone who has had any experience in opening new hospital buildings will readily understand the burden of work often entailed on those in authority who have had to bring into being a Territorial hospital and to evolve and perfect it as they went along. In the mere matter of equipment, despite the many generous helpers who were often available to assist, the task was a huge one. Everything had to be planned and provided, and the whole organisation brought into being and put upon a working basis. This is a difficult enough task when there is abundance of time and the knowledge that the patients will not arrive until everything is ready to receive them.

The Drawbacks of Premature Opening.

This latter factor has frequently been absent, and much trouble and, we fear, no little suffering and discomfort to the patient has been caused by the enthusiastic desire to open a new hospital for the reception of patients before it was humanly possible to get everything into order to receive the wounded sailors or soldiers. In our visits to the Territorial hospitals we have often been cheered and encouraged by the spirit of self-sacrifice and devotion which has been displayed by the workers. Not infrequently a suggestion that the initial stages of the work were laborious has been promptly met with the assurance that that was nothing, because "we have had to make the hospital ourselves from the beginning, and we realise that, now we have got it into order, it is our work, and we can feel proud of it on its merits." Our Generals, on their return from the Front, do themselves honour by making a point of visiting the hospitals where wounded men who have served under them are under treatment. Viscount French has been quite active in this direction, and has penetrated as far as Newcastle-on-Tyne, where he visited the 1st Northern General Hospital recently. The staff share the honour of such visits, and appreciate them greatly.

As we have already stated, we are seized with the hope that each matron and sister interested will

(*Continued at foot of page 435.*)

### THE DEPARTMENT OF NURSING—(*continued from page* 434).

co-operate with the Editor of The Hospital and do her part to make this Department of Nursing of practical use and interest, and an increasing success. It has been our invariable practice to pay for all contributions which we may publish—that is, not only articles, but items of news. The latter should occupy an important place in the scheme we have just referred to, especially when they deal with topical subjects or current events. The minimum payment, if published, is 5s. There will be no hard-and-fast rule as to space, but paragraphs of about twenty lines in length—that is to say, of 150 words—are preferred. In this section during the war we propose to give 10s. to the correspondent who may send us in any week the best incident connected with hospital life and work. In this way matrons and sisters will, we hope, receive practical aid and encouragement; while those who reciprocate will be able to render us service by making it possible successfully to continue and develop the scheme we have here outlined. Contributions should be addressed to The Editor, The Hospital, 28 & 29 Southampton Street, Strand, London, W.C., and, to be in time for the current week's issue, should reach him by the first post on Mondays.

Instruction is given to all the nurses in cooking for the sick and convalescent.

It may be mentioned that the King's College Convalescent Home at Hemel Hempstead affords the nurses an opportunity of becoming proficient in the last-named subject on the list, while at the same time giving an opportunity for refreshment to any who may be temporarily out of health in the course of their training.

### The Nurses' Quarters.

Full advantage was taken of past experience in planning the nurses' quarters at the new King's. The adaptation of means to an end is their most conspicuous feature. Every detail of the nurse's life, from the hour of rising right through the day, was taken into consideration in planning for their convenience. The result is light, air, space, privacy, compactness, order, and beauty. The latter quality, so rarely present in the modern nurses' home, has descended, as it were, unsought.

The nurses' bedrooms extend along corridors, one over another, each designed to hold twenty-eight. There are four bathrooms and lavatories to each corridor, so that a bathroom is available for every seven nurses. In addition, each corridor has its boot-room, its hair-dressing room—fitted with shampoo apparatus, a detail seldom sufficiently attended to in the provision made for nurses—and a little room for drying wet garments, which is fitted with much appreciated materials for ironing or doing repairs. In very few nurses' quarters is any provision made for the kind of occupation which makes a litter, and this little room is a concession to the working woman's needs which ought to find imitation. In the centre of each corridor hangs a clock facing both ways, with distinct figures for short-sighted eyes. The bell which rings to wake the nurses and to indicate their hours on duty and meal-times is set in motion from the matron's office, where each floor is indicated on a disc below its own number and shows a red light until turned off. By this arrangement every corridor can be called up when wanted, the night nurses having their own corridors and hours. The bedrooms are as airy, pleasant, and practically fitted up as might be guessed from their environment. The sitting-rooms are fine apartments, furnished with sure taste, and the largest one gives ample opportunities for dancing, which is a favourite recreation.

### The Time-Table.

Arrangements are made with a view to providing all members of the day staff with a period of four consecutive hours every day for exercise and recreation. These periods off duty are taken at the following hours by consecutive groups:—7 a.m. to 11 a.m.; 10 a.m. to 2 p.m.; 2 p.m. to 6 p.m.; 5 p.m. to 9 p.m. In order to facilitate this arrangement and work it in with the requirements of the wards, the probationers are divided into two groups, whose hours of rising, prayers, and meal-times are different. Some rise, for instance, at 6 a.m. and retire to rest at 9.30, while others rise at 8.30 a.m. and retire to rest at 10.30 p.m. As regards the numbers of the staff, the plan, it is explained, works out at one additional probationer to each ward. The night duty hours, usually a blot on hospital administration, have been reduced to 10¾—viz. from 8.45 p.m. to 7.30 a.m.—and three short intervals for refreshment are allowed for during the night, for tea is to be had between 11.30 and 12.30 midnight, between 3.30 and 4.30 a.m., and again, if wished, at 6.45 a.m. Once a month the night nurses get two nights off duty, leaving at 8.30 a.m., returning at 2 p.m. The undue strain of night duty is, therefore, considerably relieved.

### The Nurses' Dispensary.

Among so large a number of women, many of whom have but newly entered upon duties which make special demands on their physique, it is certain that there will always be a certain proportion in need of medical advice, if not of treatment. A special feature has been introduced to promote their comfort in the shape of a complete little dispensary and consulting-room, reserved entirely for members of the nursing staff. Here, if any slight prick or sore or rise of temperature makes its appearance, nurses can see their special doctor at the appointed hour without fuss or waste of time. This prompt attention to slight ailments is an axiom common to all hospitals, and this consulting-room enables the doctor to prescribe for and advise the staff conveniently, both to himself and to them. Good sick-rooms are provided for any who are ill.

### The Domestic Quarters.

It is now well recognised that the work of the domestic staff is so inextricably associated with the nursing that no hospital can afford to disregard the need for order, cleanliness, and refinement in the accommodation provided for this section of the household. There is matter for congratulation in the admirable organisation of this wing at Denmark Hill. In many respects the corridors for the domestics have a resemblance to those provided for the nurses. The same system of clocks and bells works for punctuality. Ample bathrooms, closets for boots and boxes, cubicles so constructed as to be practically equivalent to single bedrooms, these are features which all make for efficiency. The upper maids have separate bedrooms, and there is much to encourage a spirit of pride in the work and contentment with a service which offers good promise of promotion. We were particularly struck with the well-planned scrubbers' room on the basement floor, containing ample cupboard space, wherein each worker had a separate locker to contain all the implements of her craft. The chapel is a fine building, containing accommodation for 250 worshippers. The organ, since restored, and many parts of the old building were transferred bodily, to reappear in the new structure, notably the fine old painting which hangs over the altar. There are services on Sunday, with celebrations of the Holy Communion. Prayers are read morning and evening, and the religious life of the hospital centres, as it ought to do, round its own chapel.

## Figures and Tables

**Figure f1:**